# Improved exposure of curcumin-loaded nanocapsules: drug quantification in LPS-induced *Drosophila melanogaster* and pharmacokinetics in Wistar rats

**DOI:** 10.3389/fphar.2025.1688992

**Published:** 2025-12-01

**Authors:** Ana Pozzato Funghetto-Ribeiro, Camila de Oliveira Pacheco, Flávia Elizabete Guerra Teixeira, Joane Ferreira, Eliana Fernandes, Gustavo Guerra, Francine Johansson Azeredo, Sandra Elisa Haas

**Affiliations:** 1 Graduate Program in Biochemistry, Federal University of Pampa (Unipampa), Uruguaiana, Rio Grande do Sul, Brazil; 2 Pharmacology and Pharmacometric Laboratory, LABFAR, Federal University of Pampa (Unipampa), Uruguaiana, Rio Grande do Sul, Brazil; 3 Graduate Program in Pharmaceutical Sciences, Federal University of Santa Maria (UFSM), Santa Maria, Rio Grande do Sul, Brazil; 4 Laboratory of Pharmacological and Toxicological Evaluations Applied to Bioactive Molecules, LaftamBio Pampa, Federal University of Pampa (Unipampa), Itaqui, Rio Grande do Sul, Brazil; 5 Center for Pharmacometrics and Systems Pharmacology, CPSP, University of Florida, Orlando, FL, United States

**Keywords:** polymeric nanocapsules, NAMs, pharmacokinetics, bioavailability, drug delivery

## Abstract

**Introduction:**

Curcumin (CUR) has broad pharmacological potential; however, its clinical efficacy is hindered by low aqueous solubility, extensive presystemic metabolism, and poor oral bioavailability. Nanoencapsulation strategies have been proposed to overcome these limitations.

**Methods:**

We evaluated the exposure of poly(ε-caprolactone) nanocapsules coated with polysorbate 80 containing CUR (NC-CUR) using a validated bioanalytical approach capable of quantifying CUR in whole-body homogenates of Drosophila melanogaster and in rat plasma. Healthy and LPS-challenged flies were chronically treated with CUR or NC-CUR (37 or 110 ng/mL) for 10 days through dietary exposure. Male Wistar rats received a single intravenous (2 mg/kg) or oral (6 mg/kg) dose of CUR or NC-CUR to characterize systemic pharmacokinetics.

**Results:**

In vitro release followed a biexponential profile, with NC-CUR showing significantly prolonged release compared to free CUR (t_1/2β_ = 25.79 ± 0.87 h vs. 3.15 ± 1.37 h; *p* < 0.0001). A validated HPLC-PDA method (LLOQ = 3 ng/mL; R^2^ ≥ 0.997) enabled CUR quantification in whole flies and rat plasma. Chronic dietary exposure resulted in markedly higher CUR concentrations in flies treated with NC-CUR than free CUR (up to ∼200 vs. 75 ng/mL; *p* < 0.001), including under LPS-induced inflammatory conditions. In rats, NC-CUR increased systemic exposure following both intravenous (AUC_0-∞_: 1337.8 ± 385.2 vs. 100.4 ± 24.4 h⋅ng/mL; 13.3-fold, *p* < 0.0001) and oral administration (82.23 ± 31.68 vs. 25.55 ± 7.17 h⋅ng/mL; 3.2-fold, *p* < 0.01), reduced clearance (0.57 ± 0.18 vs. 7.71 ± 1.81 L/h; *p* < 0.0001), and accelerated absorption after oral dosing (T_max_: 0.58 ± 0.12 vs. 1.31 ± 0.28 h; *p* < 0.05).

**Discussion:**

Nanoencapsulation significantly enhanced CUR exposure in both invertebrate and mammalian systems. This cross-species analytical strategy supports *D. melanogaster* as a complementary quantitative platform for early pharmacokinetic screening and reinforces NC-CUR as a promising formulation for future translational development in inflammatory conditions.

## 1 Introduction

Curcumin (CUR) is a low molecular weight polyphenol (368.38 g/mol) extracted from the turmeric rhizome (*Curcuma longa*, *C. longa*), which is widely used as a spice in Indian cuisine and cuisine and Ayurveda medicine (Varaprasad et al., 2024). It is renowned for its broad pharmacological activities, including well-documented anti-inflammatory properties. Researchers have increasingly explored CUR’s potential in treating a variety of cancers, metabolic disorders (e.g., atherosclerosis), and neurodegenerative and autoimmune diseases (Alzheimer’s and Parkinson’s diseases) (Heger et al., 2014; Memarzia et al., 2021; Laurindo et al., 2023; Savall et al., 2024).

The primary bioactive molecules in *C. longa* are CUR and its curcuminoid derivatives, desmethoxycurcumin and bis-desmethoxycurcumin. Curcumin is regarded as the most pharmacologically potent of these, comprising approximately 70% of the total bioactive compounds. However, the physicochemical properties of CUR significantly limit its clinical application. Its chemical structure, characterized by phenolic groups and conjugated double bonds, makes it highly sensitive to environmental factors such as heat, light, and oxygen (Heger et al., 2014). Furthermore, CUR is prone to photodegradation, even in the absence of light, and is highly sensitive to light exposure and pH changes (Mondal et al., 2016). These factors pose significant challenges for large-scale use in pharmaceutical formulations. Preparing aqueous oral solutions necessitates the use of surfactants, elevated temperatures (above 37 °C), and strict light control to maintain stability (Santos et al., 2021).

Due to its lipophilic nature (Log P ∼3.2), CUR exhibits poor oral absorption in both neutral and acidic pH environments, classifying it as a Class IV compound according to the Biopharmaceutical Classification System. Its absorption improves under alkaline conditions; however, degradation also increases. Furthermore, curcumin undergoes extensive pre-systemic metabolism via both phase I and phase II reactions including reduction and conjugation (glucuronidation and sulfation), with significant interactions with macronutrients and excretion predominantly via bile and feces (Chang et al., 2023). The metabolism of CUR occurs primarily in the liver and intestine, with additional biotransformation in enterocytes and by gut microbiota, notably *Escherichia coli* (*Escherichia coli*) and *Blautia* species (Hassaninasab et al., 2011; Burapan et al., 2017).

The CUR is subject to efflux by P-glycoprotein, associated with multidrug resistance in intestinal epithelial cells, which further reduces its plasma concentrations following oral administration. Curcumin also exhibits a high binding affinity for plasma proteins, particularly albumin (Mohammadi et al., 2009). Elimination occurs primarily through the biliary route. The oral bioavailability of CUR is exceptionally low, with only about 1% of an administered dose (500 mg kg) being absorbed in rats (Yang et al., 2007). Its half-life is short, approximately 32.4 min after intravenous administration of 20 mg kg in mice (Wang et al., 2018). Despite promising preclinical outcomes in various inflammatory, autoimmune, and oncological disorders, CUR and its derivatives have not demonstrated clinical efficacy in double-blind trials, likely due to these pharmacokinetic limitations (Nelson et al., 2017).

To address these issues, employing nanocarriers such as polymeric nanocapsules (NCs) has been proposed as an effective strategy to enhance the biopharmaceutical properties of curcumin (CUR). The interfacial deposition method using a preformed polymer yields nanoparticles of subcellular dimensions, which increases the surface area available for dissolution and facilitates interaction with biological fluids (Liu et al., 2024). Additionally, the incorporation of hydrophilic surfactants on the NC surface improves wettability and dispersion in aqueous media, thereby enhancing solubility in gastric and intestinal environments (Michels et al., 2019; Liu et al., 2024). This nanoarchitecture also protects CUR from hydrolytic and photochemical degradation and promotes controlled release (Santos et al., 2021). Consequently, NCs increase systemic exposure and optimize pharmacokinetic parameters such as absorption, distribution, and elimination, ultimately improving the pharmacological response (Michels et al., 2019).

Our research group has extensively investigated the nanoencapsulation of various compounds for treating diseases such as malaria, schizophrenia, and Alzheimer’s disease (Velasques et al., 2018; Funguetto-Ribeiro et al., 2023a; Funguetto-Ribeiro et al., 2023b). Recently, we found that CUR-loaded NC (NC-CUR) exhibited therapeutic and neurorestorative potential in a rat model of Alzheimer’s disease, reducing cerebral oxidative stress and glial fibrillary acidic protein, a biomarker of astrocyte activation (Savall et al., 2024). In a *D. melanogaster* (*Drosophila melanogaster*) model, chronic treatment with NC-CUR improved survival rates and behavioral parameters, including locomotor activity, exploratory performance, and antioxidant activity, evidenced by reduced thiobarbituric acid reactive substances and reactive species levels, along with increased activity of glutathione S-transferase, superoxide dismutase, and catalase enzymes (Fernandes et al., 2023).

The use of alternative models such as *D. melanogaster*, *Caenorhabditis elegans* (*C. elegans*), and zebrafish is becoming more widespread due to their low cost, ease of handling, and homology to many human genes, making them valuable tools in preclinical research (Funguetto-Ribeiro et al., 2023b; Machado et al., 2024). These models are primarily applied to evaluate effects on survival, genetic and metabolic profiling, and the response to bioactive compounds (Sadova et al., 2024). Moreover, they present a compelling alternative to mitigate or replace the use of vertebrate animals (e.g., rodents) in preclinical studies (Martín-Peña et al., 2018; Fernandes et al., 2023). It has been shown that the intestinal physiology of *D. melanogaster* is comparable to that of humans, providing novel insights for pharmacokinetic evaluations of orally administered substances, particularly valuable for exposure assessment (Miguel-Aliaga et al., 2018).

Despite increasing interest in New Approach Methodologies (NAMs), studies assessing systemic exposure to bioactive compounds in invertebrates using robust quantitative approaches such as high-performance liquid chromatography (HPLC) remain scarce (Sadova et al., 2024). Although approximately 75% of human-related genes are conserved in *D. melanogaster* and key pharmacokinetic processes such as intestinal absorption, metabolic pathways, and xenobiotic detoxification show strong homology with mammals, current investigations in flies are predominantly limited to survival, behavioral or oxidative stress endpoints (Moraes and Montagne, 2021; Mishra et al., 2022). No study has directly compared quantitative exposure profiles obtained in *D. melanogaster* with those from rodent models, which remain the gold standard for preclinical pharmacokinetic evaluations.

To the best of our knowledge, no validated bioanalytical method has been reported for determining curcumin (CUR) systemic exposure in *D. melanogaster*, nor for establishing cross-species relationships. Given the well-recognized challenges of CUR related to its low solubility, limited stability, and poor bioavailability, the development of robust analytical strategies capable of accurately quantifying CUR in diverse biological matrices is essential for advancing both preclinical and translational research.

In this context, our study addresses a critical methodological gap by introducing, for the first time, a validated HPLC-PDA method capable of quantifying CUR in whole-body samples of *D. melanogaster* and by characterizing its pharmacokinetic behavior in parallel with healthy rodent models. These findings reinforce the value of invertebrate species as alternative organisms for early pharmacokinetic screening and support the use of *D. melanogaster* as a complementary model for evaluating *in vivo* performance of novel nanoformulations.

## 2 Experimental design

### 2.1 Chemicals and reagents

Poly-(ε-caprolactone) (MW = 80.000 g/mol), capric/caprylic triglycerides, sorbitan monostearate (Span^®^ 60), polysorbate 80, CUR (purity >75.0%, C1386), piroxicam (purity >98%, P5654), lipopolysaccharide from *E. coli* O55:B5 (L2880), and phosphoric acid were acquired from Sigma-Aldrich (United States). HPLC-Grade acetonitrile, methanol, and triethylamine were obtained from Dynamica (Brazil). Water was purified using a Milli-Q system (Millipore, United States). All other chemicals and reagents employed were of analytical grade.

### 2.2 Nanocapsule preparation

The NCs were synthesized via the interfacial deposition method of the preformed polymer, as previously described (Santos et al., 2021). The organic phase consisted of poly(ε-caprolactone) (0.1 g), curcumin (CUR, 0.006 mg), capric/caprylic triglycerides (330 µL), and Span^®^ 60 (0.0788 g) dissolved in acetone (27 mL) under magnetic stirring at 40 °C ± 2 °C. Span^®^ 60 was previously solubilized in ethanol (2 mL) at the same temperature. The aqueous phase was composed of ultrapure water (53 mL) and polysorbate 80 (0.0788 g), also kept under magnetic stirring at room temperature. The organic phase was then slowly poured into the aqueous phase under continuous stirring, and the mixture was maintained under agitation for 10 min to promote nanocapsule formation. Subsequently, the organic solvent and part of the aqueous phase were removed by rotary evaporation, resulting in a final formulation volume of 10 mL, with a CUR concentration of 0.6 mg/mL.

#### 2.2.1 Physicochemical characterization and nanocapsule stability assessment

The NCs were characterized *in vitro* by their particle size, pH, zeta potential, and drug content. The mean particle diameter and polydispersity index were measured using laser diffractometry (LD) technique (Mastersizer 2000, Malvern Instruments, United Kingdom) after diluting 10 μL of the suspension in distilled water (10 mL), with analyses performed in triplicate. The pH measurements were conducted immediately post-preparation with a calibrated potentiometer (Hanna Instruments, Brazil), also in triplicate. Zeta potential was determined by a Nanobrook 90PlusPals instrument (Brookhaven Instruments, United States) employing electrophoretic mobility, following the dilution of the sample (10 μL) in 1 mM NaCl solution (10 mL) filtered through a 0.22-μm membrane (Millipore, United States), with results presented in millivolts (mV) and obtained in triplicate. Drug content was analyzed by HPLC with photodiode array detection (PDA) following our previously established method (Santos et al., 2021).

The physicochemical stability was evaluated over 30 days, repeating the analyses on days 1, 14, 21, and 30, with the formulation stored at ambient temperature (25 °C ± 5 °C) and shielded from light.

#### 2.2.2 *In vitro* release

*In vitro* CUR release from the NCs was assessed using the dialysis bag method (25 × 16 mm, 12,000–14,000 Da molecular weight cut-off). The release medium was phosphate-buffered saline, prepared according to pharmacopeial standards and consisting of 0.02% KCl (w/v), 0.02% KH_2_PO_4_ (w/v), 0.116% Na_2_HPO_4_ (w/v), and 0.8% NaCl (w/v) (adjusted with 10 mM NaOH, pH 7.4) (Anvisa, 2019).

The CUR was formulated in a 2% polysorbate 80 aqueous solution, achieving a final concentration of 0.6 mg/mL. The dialysis bags containing CUR-loaded NCs were immersed in the phosphate-buffered saline medium at 37 °C with horizontal shaking at 50 rpm.

A free CUR solution was also evaluated in the release study to confirm that the dialysis membrane did not impede CUR diffusion. At predetermined intervals, 2 mL of the medium was withdrawn for analysis with a UV spectrophotometer (UV-1800, Shimadzu, Japan) at 427 nm. The experiments were conducted in triplicate (n = 3), with results reported as the percentage of drug released over time.

#### 2.2.3 CUR release kinetics and *in vitro* mechanism

The kinetics of CUR release were characterized by fitting the cumulative percentage of drug released over time to various mathematical models using DDSolver 1.0 (an Excel-plugin module). Evaluated models included zero-order (Equation 1), monoexponential (Equation 2), bi-exponential (Equation 3), Weibull (Equation 4), and Higuchi (Equation 5).

$$F=k_{0}\cdot t$$ (1)

$$F=100\;\cdot \left(1-e^{-k_{1\cdot }t}\right)$$ (2)

$$F=100\cdot \left(1-Ae^{-\alpha t}+Be^{-\beta t}\right)$$ (3)

$$F=100\cdot \left(1-e^{-\frac{t^{\beta }}{\alpha }}\right)$$ (4)

$$F=k_{H}\cdot t^{0.5}$$ (5)

where F is the percentage of drug released at time t (h), *k*_0_ is the zero-order release constant (h^−1^), *k*_1_ is the first-order release constant (h^−1^), A and B represent the fractions of drug distributed in different phases, and α and β (h^−1^) are the specific kinetic rate constants. *k*_H_ represents the Higuchi release constant.

The drug release mechanism was further evaluated by fitting the data to the Peppas-Sahlin (Equation 6) and Korsmeyer-Peppas (Equation 7) models:

$$F=k_{1}\cdot \;t^{0.5}+k_{2\;}\cdot t$$ (6)

$$F=k_{KP}\cdot t^{n}$$ (7)

where *k*_1_ is the constant (h^−1^) denoting the relative contribution of *t* ^0.5^ dependent drug diffusion to drug release, *k*_2_ is the constant (h^−1^) denoting the relative contribution of *t*-dependent polymer relaxation to drug release, *k*_KP_ is the kinetic constant of the Korsmeyer-Peppas model, and *n* is the diffusion exponent that indicates the drug transport mechanism.

The release profiles were plotted and fitted using different *in vitro* release models. The results of the *in vitro* drug release study were evaluated based on dissolution efficiency and independent/dependent kinetic models. Model selection was performed considering the highest correlation coefficient (*R* ^2^) and the Akaike Information Criterion (AIC) (Vieira et al., 2016).

### 2.3 Bioanalytical method validation

#### 2.3.1 Liquid chromatographic instrumentation and conditions

Chromatographic analyses were conducted using a Shimadzu (Japan) LC system equipped with a SPD-M20A PDA detector, an LC-20AT pump, a CBM-20A system controller, a DGU-20A3 degasser, and a SIL-20 autosampler. Data acquisition and processing were managed using LC Solution software (v. 1.22 SP1). Chromatographic separation was accomplished on a reversed-phase Waters C18 column (150 × 4.6 mm × 5 µm) and a pre-column containing the same material.

The mobile phase consisted of acetonitrile, methanol, and water in a ratio of 43:10:47 (v/v/v). The aqueous phase included 0.3% triethylamine, and the pH was adjusted to 3.0 with phosphoric acid. The mobile phase was filtered through a 0.45 µm membrane filter (Millipore, United States) and degassed in an ultrasonic bath for 30 min. The flow rate was set at 1 mL/min, and the column oven temperature was maintained at 30 °C. The CUR and piroxicam (internal standard [IS]) were detected at wavelengths of 424 and 365 nm, respectively, with an injection volume of 50 µL. The total run time per analysis was 11 min.

The validation procedures adhered to the Food and Drug Administration, the Brazilian National Health Surveillance Agency, and the International Council for Harmonisation of Technical Requirements for Pharmaceuticals for Human Use guidelines (FDA, 2018; ICH, 2019; Anvisa, 2024). These procedures included assessments of specificity, lower limit of quantification (LLOQ), linearity range, recovery, accuracy, precision, and stability. Additionally, evaluations of carry-over and matrix effects were conducted. The matrices analyzed were homogenized from whole body flies (*D. melanogaster*) and plasma from male Wistar rats.

#### 2.3.2 Calibration standards and quality control samples

A standard stock solution of CUR was prepared at a concentration of 200 μg/mL. An intermediate solution at 2 μg/mL was derived from this stock. Calibration standards were prepared to reach final concentrations in homogenized flies of 3, 6, 12, 24, 48, 96, and 150 ng/mL through serial dilution of the intermediate solution (n = 3). Quality control samples for evaluating precision, accuracy, and stability were prepared at CUR concentrations of 3, 48, 96, and 150 ng/mL in homogenized flies. The calibration curve for rat plasma aimed to achieve the same concentrations. For the IS, a solution of piroxicam was prepared at a concentration of 1 μg/mL, yielding a final concentration of 100 ng/mL. All standard stock solutions were stored at −20 °C until use.

#### 2.3.3 Fly and rat plasma extraction procedure

The stock solutions were followed by the preparation of a homogenate for extracting CUR from flies, using the whole body of the flies fragmented in solvent. This homogenate was prepared by weighing 0.02 g of whole flies, followed by adding 600 µL of acetonitrile and homogenizing with an Ultra-Turrax (T10, GmbH, Germany) for 30 s at 28,800 rpm. The mixture was centrifuged at 14,000 rpm for 10 min at 4 °C to obtain the whole-body fly homogenate supernatant (S1). Subsequently, 90 µL of S1 was spiked with 10 µL of each calibration standard and 10 µL of the IS. Deproteinization was achieved by adding 1,000 µL of acetonitrile and 10 µL of 0.2% HCl. The mixtures were vortexed for 5 min, then centrifuged at 14,000 rpm for 10 min at 4 °C. The supernatant was separated, evaporated to dryness using a centrifugal vacuum concentrator at 45 °C for 60 min, and the residue reconstituted in 100 µL of mobile phase.

The rat plasma extraction was processed similarly to that of the flies, with 10 µL of each calibration standard sample mixed with 90 µL of blank rat plasma and 10 µL of the IS, followed by deproteinization with acetonitrile.

#### 2.3.4 Linearity and lower limit of quantification

The calibration curve was generated for each analytical run by spiking S1 with known concentrations of CUR (3–150 ng/mL) and the IS at 100 ng/mL. The same concentration range was applied to plasma. Three standard curves, each comprising seven nominal CUR concentrations, were analyzed to confirm the method’s linearity. A linear weighted regression model (1/x^2^, x = concentration) of the peak area ratios of CUR to IS against the CUR nominal concentrations was used to determine the slope, intercept, and linearity (correlation coefficient, *R* ^2^).

The sensitivity was evaluated as LLOQ was part of the precision and accuracy evaluation. Acceptability criteria required precision within ±15% of the percentage coefficient of variation (CV%) and accuracy within ±15% deviation from nominal values. For the LLOQ, the CV% should not exceed 20% (ICH, 2019).

#### 2.3.5 Selectivity and specificity

Selectivity and specificity were assessed by comparing chromatograms of healthy flies and those with LPS-induced inflammation, spiked with known concentrations of CUR (3 and 150 ng/mL) and IS (100 ng/mL) in triplicate. This ensured that no interfering substances from the inflammatory model affected the quantification of CUR and IS, evidencing no significant peaks at their retention times in blank samples. Furthermore, plasma selectivity and specificity were evaluated using blank rat plasma, alongside blank lipemic and hemolyzed rat plasma, compared with blanks spiked with 3 and 150 ng/mL of CUR and 100 ng/mL of the IS, ensuring no endogenous substances interfered with CUR and IS detection.

#### 2.3.6 Residual effect

The potential for carry-over effects was evaluated by injecting the highest concentration of CUR (150 ng/mL) into the HPLC-PDA system (n = 3). Subsequently, blank S1 samples were analyzed under identical chromatographic conditions, with three injections: one prior and two immediately following the high concentration injection. Similarly, the highest concentration of CUR (150 ng/mL) was injected into the rats, followed by the analysis of plasma samples under the same conditions. This ensured the absence of residual CUR in the system that could interfere with subsequent analysis. The results were considered acceptable if they did not exceed 20% of the LLOQ for CUR and 5% for the IS (FDA, 2018; ICH, 2019).

#### 2.3.7 Matrix effect

The matrix effect was evaluated by comparing the peak area ratios of CUR and the IS in spiked fly S1 and blank rat plasma samples with those in a pure solution at concentrations of 3, 48, and 150 ng/mL (n = 3). This assessment confirmed that endogenous substances in both the plasma and fly S1 samples do not interfere with the accurate quantification of CUR and the IS. The results were compared across samples in solution at identical concentrations using the normalized matrix factor (NMF), whereas the criterion of acceptance was CV <15% (FDA, 2018; ICH, 2019):

$$NMF=\frac{Analyte\;area\;in\;matrix\;/\;IS\;area\;in\;matrix}{Mean\;analyte\;solution\;area\;/\;mean\;IS\;solution\;area}$$

#### 2.3.8 Recovery

The CUR recovery was assessed at concentrations of 3, 48, and 150 ng/mL in both fly S1 and rat plasma samples (n = 3). Recovery rates were determined by comparing the peak area ratios of CUR in spiked samples post-extraction to those in non-extracted standards at identical concentrations. For each matrix, three blank samples were used. Results were expressed as the recovery percentage, reflecting the extraction efficiency at each concentration level (ICH, 2019).

#### 2.3.9 Precision and accuracy

Precision and accuracy, also referred to as bias, were evaluated by analyzing three samples of S1 and rat plasma spiked with CUR at four concentrations (3, 48, 96, and 150 ng/mL) over three separate validation days. Precision was assessed by calculating the %CV for each concentration, while accuracy was determined by the mean percent error between the measured and nominal concentrations. In all instances, values were expected to be within 15% of the nominal concentrations, except at the LLOQ, where deviations should not exceed 20% (FDA, 2018; ICH, 2019).

#### 2.3.10 Stability experiments

The stability of CUR’s standard stock solution (3 and 150 ng/mL) was evaluated at −20 °C and −80 °C for both fly and rat plasma samples, respectively. Stability tests were performed under identical conditions for both matrices: room temperature for 6 h (22 °C ± 3 °C), in the autosampler for up to 24 h (22 °C ± 3 °C), after 60 days in a freezer (−80 °C ± 2 °C), and across three freeze-thaw cycles, with each thawing period at room temperature for approximately 2 h and refreezing for 12 h at −20 °C. For each species, three replicates at concentrations of 3 and 150 ng/mL were tested.

Stability was assessed by comparing the peak area ratio of CUR to the IS in the test samples against freshly prepared standards of the same concentrations for both fly and rat plasma samples. Samples were deemed stable if the results fell within the acceptable limits of accuracy (±15% of nominal concentration) and precision (±15% CV). For the LLOQ, the CV was not to exceed 20% (FDA, 2018; ICH, 2019).

### 2.4 *In vivo* study

#### 2.4.1 *D. melanogaster* culture and CUR exposure

The *D. melanogaster* flies (Harwich strain) were sourced from the Laftambio Laboratory at the Federal University of Pampa (Itaqui, Rio Grande do Sul, Brazil). The flies were maintained in glass flasks under controlled conditions: a 12-h light/dark cycle, at a temperature of 25 °C ± 1 °C, and 60%–70% humidity. Their diet comprised 76.59% corn flour, 8.51% wheat germ, 7.23% sugar, 7.23% milk powder, 0.43% salt, and 0.08% methylparaben (Fernandes et al., 2023).

**FIGURE 1:**
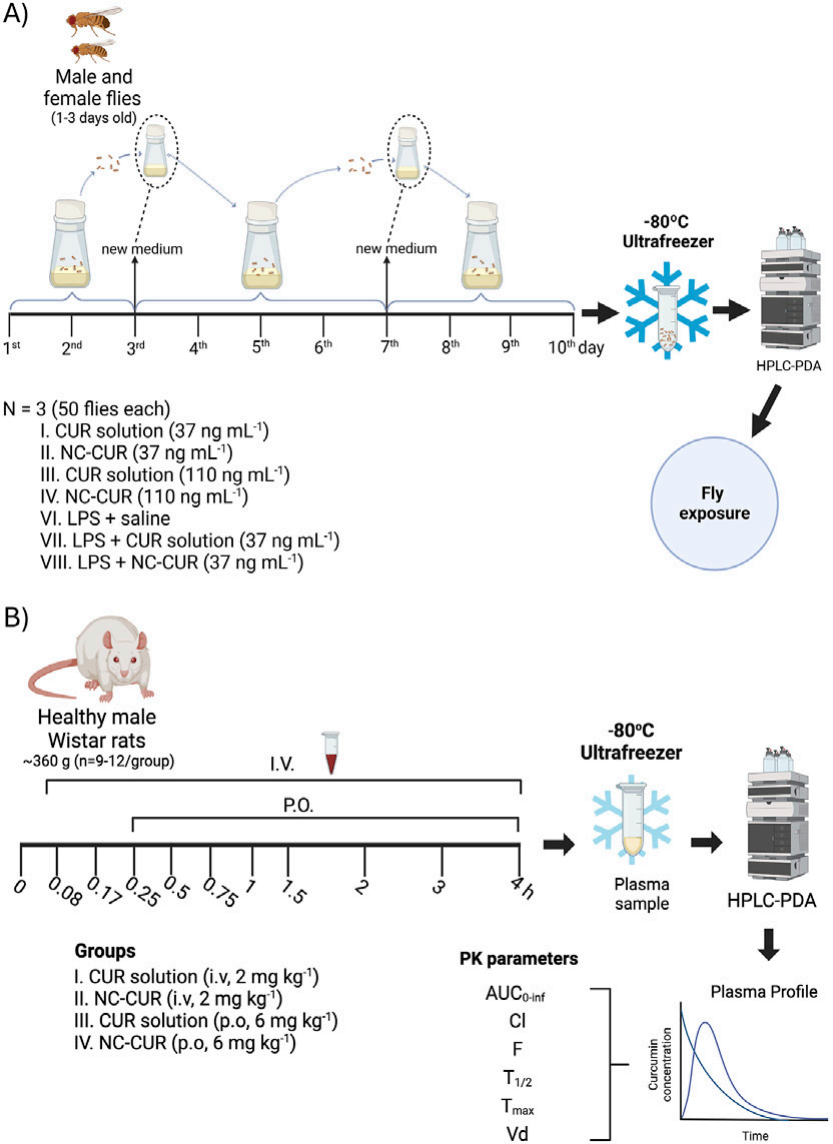
**(A)** CUR Exposure and sample collection in *D. melanogaster*. Healthy flies were exposed to CUR or NC-CUR in their food medium at concentrations of 37 and 110 ng/mL for 10 days, with food replaced on days 3 and 7. Additional groups were exposed to LPS in the food medium, combined with CUR or NC-CUR treatments. After 10 days, flies were euthanized, and whole bodies were quantified by HPLC-PDA. **(B)** Pharmacokinetic study design and sample collection timeline. The pharmacokinetics were evaluated in male Wistar rats following a single intravenous bolus dose of 2 mg/kg or a single oral dose of 6 mg/kg. Blood samples were collected at predetermined times, processed to obtain plasma, and analyzed using HPLC-PDA to determine CUR concentrations and pharmacokinetic parameters.

Adult healthy flies (1–3 days old) of both sexes were used, with each group consisting of 25 males and 25 females (n = 6 replicates/50 flies each). The flies were exposed to CUR by incorporating either CUR solution or NC-CUR into their food medium at concentrations of 37 and 110 ng/mL for 10 days (Figure 1A). The medium was replaced every 3 days. The dosing and study design were based on our previous study (Fernandes et al., 2023). After 10 days, the flies were collected into microcentrifuge tubes, euthanized on ice, and their bodies stored at −20 °C until CUR quantification via HPLC-PDA. The experimental groups were as follows: (i) saline (negative control); (ii) CUR solution, 37 ng/mL; (iii) CUR solution, 110 ng/mL; (iv) NC-CUR, 37 ng/mL; and (v) NC-CUR, 110 ng/mL. The results are presented as the mean CUR concentration in flies’ homogenate ±standard deviation (SD).

##### 2.4.1.1 Lipopolysaccharide-induced inflammation in *D. melanogaster* and CUR exposure

A model of generalized and progressive inflammation was induced through chronic exposure to lipopolysaccharide (LPS) (250 μg/mL) in the food medium over 10 days, combined with treatment using CUR or NC-CUR at 37 ng/mL. Each group consisted of 25 males and 25 females (n = 3 replicates/50 flies each), divided as follows: (i) LPS + saline; (ii) LPS + CUR solution; (iii) LPS + NC-CUR (Figure 1A). The medium was replaced every 3 days, maintaining the same CUR and LPS concentrations as described (John et al., 2022; Fernandes et al., 2023). After 10 days, the flies were collected, euthanized on ice, and their bodies stored at −20 °C for subsequent quantification of CUR by HPLC-PDA. Results are expressed as the mean CUR concentration ±SD in flies’ homogenates.

#### 2.4.2 Pharmacokinetic study in rodents

This study was approved by the institution’s Animal Care and Use Committee (protocol number 02/2023). Male Wistar rats (60 days old, weighing 320–400 g) were housed under a controlled 12-h light/dark cycle during the acclimation period, with unlimited access to food and water. All animal procedures adhered to the guidelines of the Principles of Laboratory Animal Care (National Research Council, 2011).

Healthy male rats were administered CUR intravenously (2 mg/kg) or orally (6 mg/kg) (n = 9–12 animals per group). The experimental groups were divided as follows: (i) CUR solution, intravenously (iv); (ii) CUR solution, orally (po); (iii) NC-CUR, iv; (iv) NC-CUR, po (Figure 1B). The CUR solution was prepared by dissolving CUR in water with 2% polysorbate 80 at 37 °C to a concentration of 0.6 mg/mL. As described in our previous study, the NC-CUR was prepared via the interfacial deposition method of preformed polymers, ensuring the same final concentration (Santos et al., 2021).

After administration, blood samples were collected via lateral tail vein puncture into heparinized tubes at predetermined time points (0.083, 0.17, 0.25, 0.5, 0.75, 1, 1.5, 2, 3, and 4 h). For the oral administration groups, blood sampling began 0.25 h post-gavage (Figure 1B). Plasma was separated by centrifugation at 12,000 rpm for 10 min at 4 °C ± 1 °C and stored at −80 °C until analysis by HPLC-PDA. Results were expressed as the mean plasma concentration ±SD of CUR in ng/mL.

Pharmacokinetic parameters were calculated using non-compartmental analysis with PKanalix 2023R1 software (Lixoft, France). Plasma concentration-time profiles for each experimental group were analyzed individually.

### 2.5 Statistical analysis

Statistical analysis was performed using GraphPad Prism software (v. 8). Data are presented as mean ± SD. Comparisons between groups were made using either the Student’s t-test, one-way analysis of variance (ANOVA), followed by Tukey’s *post hoc* test when applicable or two-way ANOVA, followed by Sidak’s multiple comparisons test. A *p*-value <0.05 was considered statistically significant.

## 3 Results and discussion

### 3.1 Nanocapsule physicochemical and stability evaluation

The initial physicochemical parameters were consistent with earlier reports from our research group, indicating the reproducibility of our preparation method (Santos et al., 2021). Furthermore, the physicochemical characterization of the NCs over 30 days demonstrated their adequate stability across most evaluated parameters, though some statistically significant variations over time were observed *in vitro* (Table 1).

**TABLE 1:** Physicochemical characterization and stability of NC-CUR.

Parameter	D1	D14	D21	D30
D_(4,3)_ (nm)	198 ± 1	194 ± 1	191 ± 1	193 ± 1
SPAN	1.64 ± 0.06	1.63 ± 0.03	1.59 ± 0.06	1.60 ± 0.01
Zeta potential (mV)	−15.14 ± 0.34	−17.04 ± 0.82	−19.1 ± 0.8	−19.14 ± 0.77
pH	5.83 ± 0.02	5.25 ± 0.07	5.29 ± 0.03	5.03 ± 0.02^*^
Drug content (%)	100.2 ± 0.7	101.3 ± 0.4	99.83 ± 0.7	98.73 ± 0.55

The results are expressed as mean ± standard deviation (SD) from three independent replicates. Statistical analysis was performed using one-way ANOVA, followed by Tukey’s *post hoc* test. D1, D14, D21, and D30 correspond to days 1, 14, 21, and 30, respectively. D_(4,3)_ represents the volume-weighted mean diameter by laser diffraction (LD), and SPAN, indicates the particle size distribution.

^a^ denotes a statistically significant difference compared to day 1 (*p* ≤ 0.05).

The D_(4,3)_ values remained relatively constant throughout the study, ranging from 198 ± 1 nm on day 1 to 193 ± 1 nm on day 30, without statistically significant differences between time points (Supplementary Figure S1). This consistency indicates a stable particle size. Similarly, the SPAN values showed minimal variation, decreasing from 1.64 ± 0.06 on day 1 to 1.60 ± 0.01 on day 30, further supporting the homogeneity of the particle size distribution over time.

The zeta potential exhibited a progressive decrease over the 30-day period, from −15.14 ± 0.34 mV on day 1 to −19.14 ± 0.77 mV on day 30. Although this decrease was statistically significant, the negative zeta potential values indicate that the NCs maintained adequate colloidal stability. Conversely, the pH of the suspensions significantly decreased from 5.83 ± 0.02 on day 1 to 5.03 ± 0.02 on day 30 (*p* ≤ 0.05), suggesting potential medium acidification over time, possibly due to changes in formulation components or excipient degradation (Bartnikowski et al., 2019).

The drug content within the NCs remained close to 100% throughout the study, ranging from 100.2% ± 0.7% on day 1 to 98.73% ± 0.55% on day 30, with no significant changes, indicating efficient drug retention within the NC.

Overall, these results demonstrate that the NC formulation remained physically and chemically stable over the 30-day evaluation period. Though minor variations in zeta potential and pH were observed, these did not compromise the encapsulation efficiency or particle size stability, reinforcing the formulation’s robustness. Additionally, incorporating CUR into the formulation may contribute to its stability, as our findings indicate that significant changes in particle size, surface charge, and pH observed in drug-free systems were not observed (Santos et al., 2021).

### 3.2 *In vitro* release

In recent years, there has been significant growth in the discovery and development of poorly soluble compounds, primarily classified under Biopharmaceutical Classification System Classes II and IV. This solubility limitation greatly affects their *in vivo* efficacy, with approximately 40% of marketed drugs falling into these categories (Kalepu and Nekkanti, 2015; Thakore et al., 2021). The *in vitro* release test is crucial for predicting drug kinetics in physiological fluids, based on the premise that for a compound to exert its therapeutic effect or be absorbed, it must first demonstrate adequate solubility and release in the tested medium (D’Souza, 2014).

**FIGURE 2:**
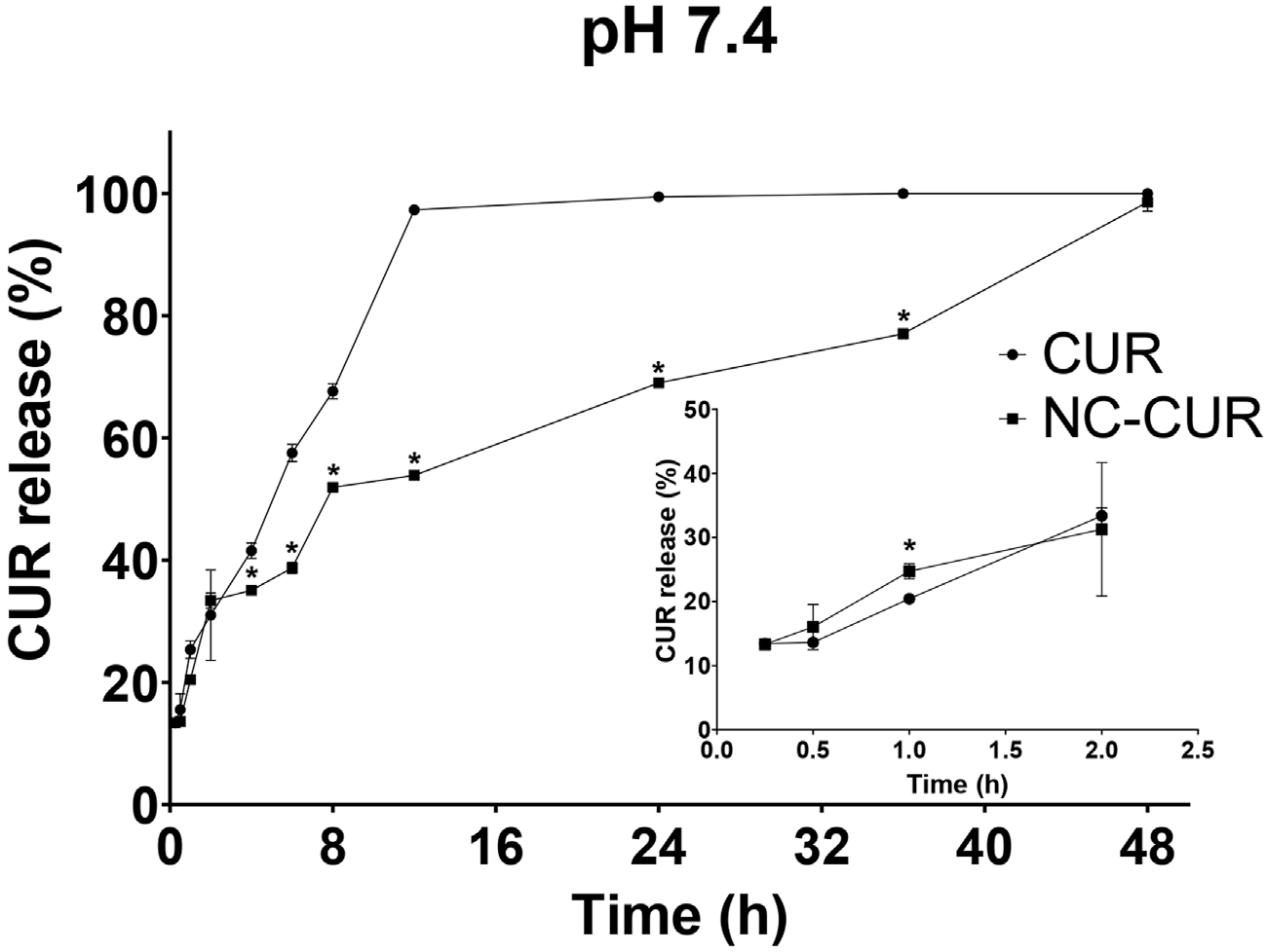
*In vitro* release profiles of free curcumin and curcumin-loaded nanocapsules in phosphate-buffered saline (pH 7.4) over 48 h. Data are expressed as mean ± standard deviation (n = 3 independent replicates). *p < 0.05 versus free CUR at the corresponding time point (two-way repeated-measures ANOVA followed by Sidak’s multiple comparisons test).

The release profiles of CUR and NC-CUR were evaluated *in vitro* over 48 h, revealing significant differences between the two formulations (Figure 2). In the initial phases, from 0.25 to 2 h, both formulations exhibited comparable release rates, with values ranging from 13.27% to 38.65% for CUR and from 13.27% to 34.29% for NC-CUR. However, after 2 h, CUR showed a rapid increase in release, reaching 56.09% at 6 h and 97.69% at 12 h. In contrast, NC-CUR demonstrated a slower, controlled release, with 52.08% at 8 h and 68.84% at 24 h.

The most pronounced difference occurred between 12 and 36 h: while CUR reached its maximum release (∼100%) at 12 h, NC-CUR continued to release drug gradually, reaching 98.94% after 48 h. These results clearly indicate that nanoencapsulation significantly slows CUR’s release rate, leading to prolonged drug release compared to the free CUR.

The *in vitro* release kinetics of both CUR and NC-CUR were evaluated using various mathematical models (Table 2). The biexponential model was determined to be the best fit based on the highest *R* ^2^ and lowest AIC values for both formulations, along with the highest MSC. Other models, such as the zero-order and monoexponential models, provided lower *R* ^2^ values (0.74 and 0.95 for CUR and 0.84 for NC-CUR), indicating less accurate fits. The biphasic release described by the biexponential model indicates an initial rapid release phase followed by a slower, sustained release phase. For CUR, the initial fast release phase was characterized by t_1/2 α_ = 3.50 ± 1.52 h, while for NC-CUR, it was significantly faster, with t_1/2 α_ = 1.27 ± 0.12 h (*p* < 0.05). The slower release phase, t_1/2 β_, showed a substantial difference between CUR (3.15 ± 1.37 h) and NC-CUR (25.79 ± 0.87 h, *p* < 0.0001), highlighting the prolonged release behavior of NC-CUR. Moreover, the higher B value for NC-CUR (60.65 ± 1.87, *p* < 0.0001) supports the observed prolonged release.

**TABLE 2:** *In vitro* release kinetics of CUR and NC-CUR obtained by fitting different mathematical models to the release profile in phosphate-buffered saline (PBS, pH 7.4).

Model	CUR	NC-CUR
Zero order		
*k*_0_ (h^−1^)	2.57 ± 0.01	1.745 ± 0.01
*R* ^2^	0.74 ± 0.01	0.84 ± 0.00
MSC	0.84 ± 0.02	1.34 ± 0.01
AIC	67.53 ± 0.24	56.35 ± 0.09
Monoexponencial		
*k* (h^−1^)	0.14 ± 0.00	0.128 ± 0.00
*R* ^2^	0.9520 ± 0.0003	0.84 ± 0.01
MSC	2.54 ± 0.01	1.34 ± 0.07
AIC	54.00 ± 0.15	56.39 ± 0.59
Biexponencial^a^		
A	78.99 ± 2.50	32.71 ± 2.04^****^
B	11.93 ± 2.50	60.65 ± 1.87^****^
t_1/2_ α (h)	3.50 ± 1.52	1.27 ± 0.12^*^
t_1/2_ β (h)	3.15 ± 1.37	25.79 ± 0.87^****^
α (h^−1^)	0.15 ± 0.02	0.55 ± 0.07^**^
β (h^−1^)	0.17 ± 0.01	0.03 ± 0.00^**^
*R* ^2^	0.99 ± 0.00	1.00 ± 0.00
MSC	3.06 ± 0.33	4.30 ± 0.32
AIC	49.80 ± 2.51	32.68 ± 2.63
Weibull		
t_1/2_ α (h)	0.15 ± 0.00	0.17 ± 0.01
t_1/2_ β (h)	0.84 ± 0.01	1.40 ± 0.01
α (h^−1^)	4.73 ± 0.06	4.05 ± 0.01
β (h^−1^)	0.82 ± 0.01	0.49 ± 0.00
*R* ^2^	0.95 ± 0.01	0.99 ± 0.00
MSC	2.58 ± 0.12	4.11 ± 0.31
AIC	53.64 ± 1.10	34.16 ± 2.56
Higuchi		
k_H_ (h^−1^)	18.05 ± 0.07	11.92 ± 0.07
*R* ^2^	0.89 ± 0.00	0.96 ± 0.00
MSC	1.74 ± 0.04	2.74 ± 0.04
AIC	60.39 ± 0.47	45.13 ± 0.37
Korsmeyer-Peppas		
*k*_KP_ (h^-n^)		22.89 ± 0.05
*n*		0.35 ± 0.001
*R* ^2^		0.98 ± 0.00
MSC		3.31 ± 0.12
AIC		40.52 ± 1.06
Peppas-Sahlin		
*k*_1_		23.86 ± 0.08
*k*_2_		−1.72 ± 0.02
*R* ^2^		0.9891 ± 0.00
MSC		3.80 ± 0.30
AIC		36.65 ± 2.49

The results were expressed as means ± standard deviations (SD) based on three independent replicates.

^a^ Chosen model between zero, mono and biexponential models.

*p-value <0.05, **p-value <0.01, ***p-value <0.001, and ****p-value <0.0001 compared to the CUR (unpaired Student’s t-test).

The Weibull and Higuchi models demonstrated reasonable fits, with *R* ^2^ values ranging from 0.95 to 0.99. NC-CUR exhibited slower diffusion and release compared to CUR, with a diffusion constant of 11.92 ± 0.07 h^−1^ for NC-CUR and 18.05 ± 0.07 h^−1^ for CUR. This difference confirms the controlled release effect conferred by the NC structure. The Korsmeyer-Peppas model (Table 2) was employed to further investigate the mechanism behind NC-CUR’s release. Free drugs typically dissolve quickly, especially in an appropriate medium such as a buffer that simulates physiological conditions. The release primarily occurs through dissolution and simple diffusion, highlighting the role of a polymeric system in controlling the release rate.

The diffusional exponent n signifies the predominant mechanism of drug release, aiding in distinguishing between release predominantly through diffusion across the polymer matrix or through a combination of diffusion and polymer relaxation. Polymer swelling might facilitate diffusion, whereas polymer erosion or dissolution could enhance controlled drug release (Kanungo et al., 2013). The diffusional exponent n = 0.35 (*p* < 0.001) suggests a Fickian diffusion-controlled release for CUR from the NC, with an *R* ^2^ value of 0.98, supporting the primacy of diffusion in controlling the release. By incorporating sorbitan monostearate into the organic phase, an organogel is formed (Santos et al., 2021). In this system, drug release is directly impacted by the viscosity of both the lipid core and the polymeric wall, thereby influencing diffusion and the controlled release profile. Our previous studies have shown that within the NC, CUR predominantly distributes in the lipid core and at the interface, irrespective of the coating type used (Nakama et al., 2020; Santos et al., 2021). As a result, diffusion through the polymer is impeded when dispersion occurs in the core. pH and Log D are essential parameters for predicting this distribution, and we found that at pH values below 8, the Log D of CUR remains constant, causing CUR molecules to concentrate in the lipid core, given its high lipophilicity (Log P ∼3.2) (Nakama et al., 2020).

Additionally, the Peppas-Sahlin model provided insights into the relative contributions of diffusion and polymer relaxation, indicating that diffusion is the dominant mechanism in CUR’s release from NC-CUR, with *k*_1_ = 23.86 ± 0.08 and a negative *k*_2_ = −1.72 ± 0.02, indicating a minor contribution from polymer relaxation. The high *R* ^2^ (0.9891) and model selection criteria (MSC) (3.80) values for this model further confirm diffusion’s primary role in CUR’s sustained release from NC-CUR.

The observed sustained release of NC-CUR holds significant therapeutic potential, enabling more stable plasma concentrations over time, which may reduce the need for frequent dosing and minimize concentration spikes that could lead to adverse effects (Laurindo et al., 2023; Tiatragoon et al., 2024). This gradual release is advantageous for long-term therapies, potentially improving treatment efficacy and patient adherence.

### 3.3 Method development and validation

The development of the HPLC-PDA method for quantifying CUR and IS in *D. melanogaster* and rat plasma were centered on optimizing separation conditions to achieve the required sensitivity, specificity, and reproducibility. This process involved testing various columns, organic solvents, and their proportions, as well as different pH levels. The optimal separation conditions were found using a mobile phase composed of acetonitrile, methanol, and water (43:10:47, v/v/v).

The aqueous phase, with a pH adjusted to 3, included 0.3% triethylamine, acidified with phosphoric acid. Triethylamine is advantageous when used in C18 columns where free silanol groups exist. These groups can ionize and strongly interact with amine-containing molecules (NH_2_ groups), resulting in undesirable tailing in chromatographic peaks (Calabuig-Hernández et al., 2016; Stoll, 2023). Triethylamine’s temporary interaction with these ionized silanol groups prevents further interactions with compounds, thereby enhancing peak shapes (Stoll, 2023).

Linearity assessment for both fly S1 and rat plasma samples across seven concentrations, ranging from 3 to 150 ng/mL, confirmed the method’s reliability; this range was derived from prior research findings. The calibration curves for both sample types showed excellent linearity, with *R* ^2^ values of 0.9979 for flies (equation y = 0.0225× + 0.4476) and 0.9998 in rats (equation y = 0.0406× + 0.0622). The LLOQ for both sample types was established at 3 ng/mL, marking the minimum concentration measurable with up to 20% coefficient of variation for precision and accuracy) (ICH, 2019; Anvisa, 2024).

**FIGURE 3:**
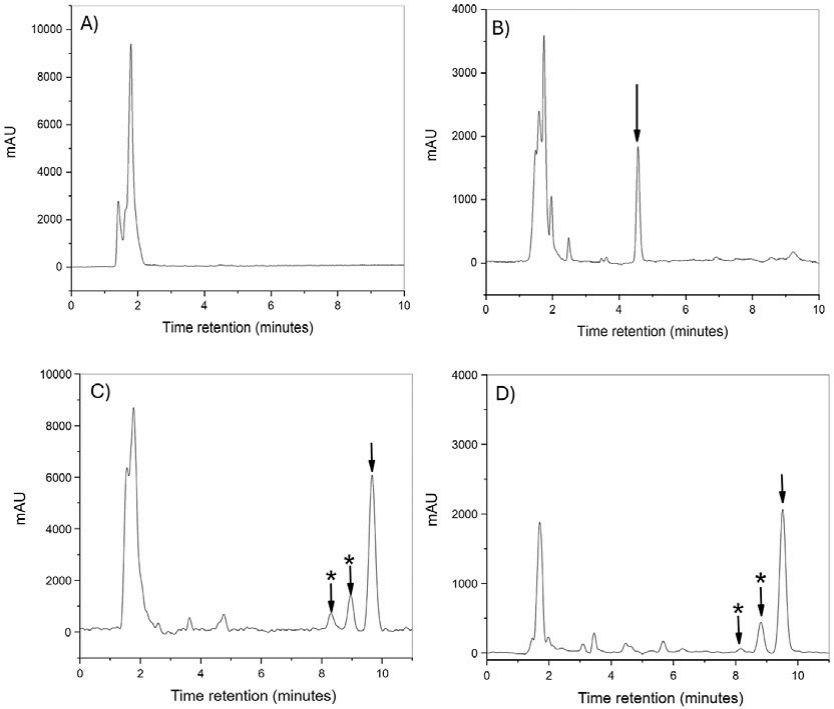
Representative HPLC-PDA chromatograms of CUR S1 flies. **(A)** Blank S1 flies (424 nm); **(B)** spiked SI at 100 ng/mL; **(C)** spiked CUR S1 at 48 ng/mL; **(D)** NC-CUR feeding medium of 110 ng/mL for 10 days (171 ng/mL); arrows indicate the analyte peaks; arrows with asterisks indicate the peaks of curcuminoids.

The developed method demonstrated highly sensitive and specific for CUR detection in biological samples from *D. melanogaster* and rats. Chromatograms of blank fly S1 samples (Figure 3A) evidenced no interfering signals at the CUR retention times, confirming the method’s specificity. The IS was consistently detected with a retention time of 4.76 min (Figure 3B). Figure 3C shows three distinct peaks were observed between 8 and 10 min in samples spiked with CUR (48 ng/mL), corresponding to curcuminoids and CUR. The peaks at 8.24 and 8.91 min represent curcuminoids, while the larger peak at 9.88 min corresponds to CUR (Schiborr et al., 2010). This result aligns with expectations, given that the CUR used in this study has a reported purity below 80%, according to the manufacturer.

Figure 3D presents chromatograms from flies exposed to NC-CUR in their feeding medium (110 ng/mL) over 10 days displayed an S1 concentration of 172 ng/mL. Additionally, samples from flies with LPS-induced systemic inflammation showed no interfering signals, underscoring the method’s robustness under different treatment conditions. Recovery rates were excellent, with 106.84% ± 6.43% and 98.66% ± 10.06% achieved at 3 and 150 ng/mL, respectively.

**FIGURE 4:**
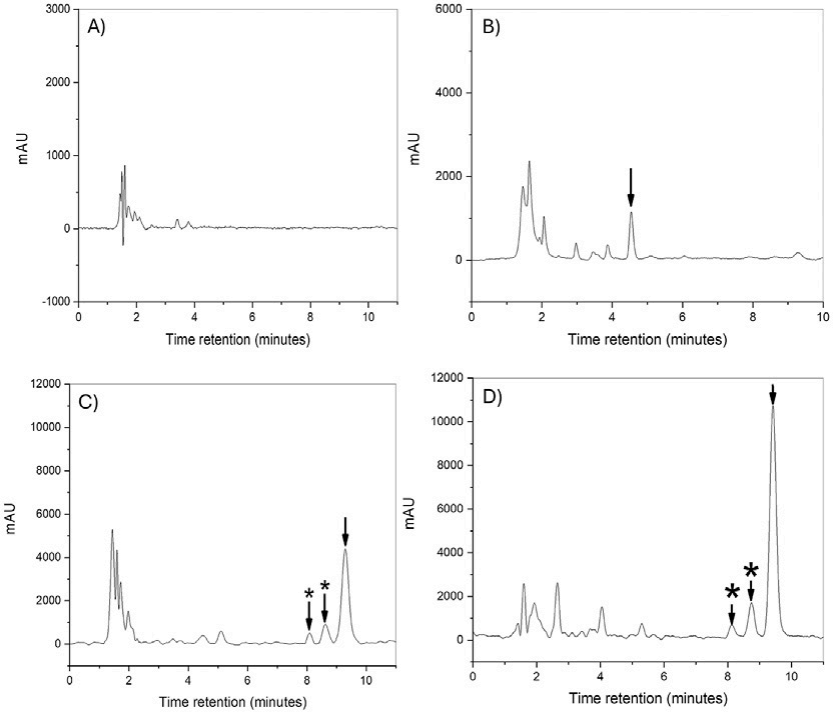
Representative HPLC-PDA chromatograms of CUR in rats. **(A)** Blank plasma (424 nm); **(B)** spiked internal standard plasma at 100 ng/mL; **(C)** spiked CUR plasma at 48 ng/mL; **(D)** NC-CUR intravenous bolus dose of 2 mg/kg (837.45 ng/mL). Arrows indicate the peaks of the analytes; arrows with asterisks indicate curcuminoid peaks.

As observed in flies, Figures 4A,B depict representative chromatograms of blank rat plasma samples at wavelengths of 424 and 365 nm, respectively, which correspond to the maximum absorbance signals for CUR and the IS. No interfering signals were observed at the analytes’ retention times, confirming the specificity of the method in rat plasma. The IS consistently exhibited a retention time of 4.81 min.

Figure 4C illustrates chromatograms of spiked plasma samples (48 ng/mL), where curcuminoids and CUR were detected between 8 and 10 min. Figure 4D presents chromatograms from rats treated with NC-CUR intravenously, resulting in plasma CUR concentrations of 837.45 ng/mL.

The presence of lipemia and hemolysis in plasma samples did not interfere with the detection of analytes. The method demonstrated high recovery rates in lipemic samples, with values of 103.41% ± 0.18% and 93.09% ± 2% at concentrations of 3 and 150 ng/mL, respectively. Recoveries in hemolyzed samples were comparable, with values of 104.29% ± 0.35% and 91.04% ± 1.43% at the same concentrations.

These findings confirm the reliability and robustness of the developed bioanalytical method for CUR quantification in biological matrices, thereby reinforcing its applicability in various sample types and experimental conditions.

The evaluation of pre- and post-spiked samples in both matrices demonstrated satisfactory recovery rates. In S1 flies, the recovery was 95.65% ± 7.51%, 104.52% ± 7.47%, and 104.27% ± 10.63% at concentrations of 3, 48, and 150 ng/mL, respectively, with NMF values of 0.99% ± 0.62%, 0.97% ± 4.99%, and 1.05% ± 9.21% were observed, respectively. Similar results were obtained in rat plasma samples, showing recovery rates of 104.01% ± 0.41%, 89.38% ± 3.79%, and 101.42% ± 1.31% for the same concentrations of 3, 48, and 150 ng/mL, respectively, with corresponding NMF of 1.15% ± 9.39%, 0.84% ± 4.27%, and 1.01% ± 0.41%.

No carry-over effects were detected in either matrix following the injection of high concentrations of CUR or the IS, as confirmed by subsequent analyses of blank samples. The precision and accuracy of the method were assessed at four different concentrations, detailed in Table 3. For fly samples, intraday precision (repeatability) exhibited a CV of 0.09%–14.1%, and intraday precision varied of 2.72%–12.94%. The accuracy for fly samples varied by 90.85%–112.37%. Similarly, the repeatability in rat plasma samples showed a CV of 0.66%–10.9%, while interday precision varied (5.72%–8.09%). The accuracy for plasma samples also varied (85.88%–100.98%), indicating consistent performance across the concentration range tested.

**TABLE 3:** Precision and Accuracy of CUR validation in spiked plasma rats and S1 flies.

Nominal concentration (ng/mL)	CUR concentration in S1 Flies (ng/mL)					CUR concentration in rat plasma (ng/mL)			
	Day	Mean	SD	CV (%)	Accuracy (%)	Mean	SD	CV (%)	Accuracy (%)
Intra-day precision^a^									
3	1	2.8	0.39	14.1	93.29	2.58	0.2	7.65	85.88
	2	3.33	0.29	8.83	110.93	49.94	5.33	10.67	104.05
	3	3.08	0.42	13.73	102.55	96.19	10.49	10.9	100.2
48	1	43.85	2.39	5.44	91.35	149.5	13.57	9.08	99.67
	2	46.55	1.26	2.71	96.97	2.6	0.15	5.67	86.76
	3	43.61	0.24	0.54	90.85	47.87	1	2.09	99.72
96	1	95.11	2.92	3.07	99.08	96.94	0.64	0.66	100.98
	2	94.93	2.43	2.56	98.89	149.57	1.01	0.68	99.71
	3	98.63	0.09	0.09	102.74	2.71	0.26	9.43	90.33
150	1	159.53	14.35	9	106.36	46.37	4.63	9.99	96.61
	2	168.55	4.98	2.95	112.37	94.11	5.16	5.49	98.03
	3	146.85	4.86	3.31	97.9	145.35	9.18	6.31	96.9
Inter-day precision^b^									
3		3.07	0.4	12.94		2.63	0.19	7.13	
48		44.67	1.96	4.38		48.06	3.89	8.09	
96		96.22	2.62	2.72		95.75	5.99	6.26	
150		158.31	12.36	7.81		148.14	8.47	5.72	

The results were expressed as means ± standard deviations (SD), followed by coefficients of variation (CV%).

^a^ Means derived from three replicates;

^b^ Means derived from nine samples.

These results meet the stringent criteria required for reliable quantification in plasma and fly matrices, with the CV or deviation from the nominal content being up to 20% for the LLOQ and up to 15% for other concentrations. This confirms that the analytical method employed was precise and accurate across the entire concentration range tested, meeting the stringent criteria required for reliable quantification in plasma and fly matrices.

The stability of CUR was assessed under several conditions, including exposure to room temperature for 6 and 24 h at 22 °C ± 3 °C, repeated freeze-thaw cycles, and long-term storage for 60 days at −80 °C (Table 4). These conditions were chosen to replicate common scenarios during sample preparation, sequential analysis, and the period between sample collection and HPLC processing.

**TABLE 4:** Stability study of CUR in spiked plasma rats and S1 flies.

Nominal concentration (ng/mL)	Stability condition	S1 Flies			Plasma rats		
		Mean ± SD (ng/mL)^a^	CV (%)	Accuracy (%)^b^	Mean ± SD (ng/mL)^a^	CV (%)	Accuracy (%)^b^
3	6 h (bench-top)	2.74 ± 0.39	14.42	84.45	2.58 ± 0.04	1.57	88.23
	24 h 6 h (bench-top)	3.23 ± 0.24	7.64	114.19	2.94 ± 0.19	6.54	98.08
	60 d (−80 °C)	3.09 ± 0.26	8.5	103.03	2.58± 0.05	2.05	86.28
	Freeze-thaw cycles	3.20 ± 0.27	8.65	106.81	2.58 ± 0.04	1.57	86.23
150	6 h (bench-top)	144.26 ± 4.68	3.24	94.59	148.64 ± 1.35	0.91	99.1
	24 h (autosampler)	142.32 ± 4.62	3.24	89.72	149.75 ± 0.92	0.61	99.84
	60 d (−80 °C)	147.59 ± 16.88	11.44	98.39	148.26 ± 0.17	0.12	98.84
	Freeze-thaw cycles	143.99 ± 13.47	9.35	95.99	148.64 ± 1.35	0.91	99.1

The results were expressed as means ± standard deviations (SD) based on three independent replicates, followed by coefficients of variation (CV%).

In S1 fly samples, the methodology demonstrated consistent performance, with CV values and accuracy falling within the acceptable ranges delineated by regulatory standards. These standards recommend a CV within 20% for the LLOQ and 15% for other concentrations, with accuracy rates in a similar range (FDA, 2018; ICH, 2019). Likewise, in rat plasma samples, the CV varied by 0.61%–6.54%, and accuracy by 88.23%–99.84%. These metrics indicate that the method remained reliable under all tested conditions, with the CV sustaining low levels at the nominal concentration of 150 ng/mL. These findings show that the analytical method is stable and reliable under the assessed conditions, thus supporting its application in pharmacokinetic and bioanalytical studies for accurate quantification of CUR in both fly S1 and plasma matrices is required.

### 3.4 *In vivo* study

#### 3.4.1 Fly whole-body CUR quantification

The dose selection for *D*. *melanogaster* treatment was based on our previous findings, in which concentrations ranging from 10 to 300 μM (equivalent to 3.7–110.5 ng/mL) produced increased survival and enhanced antioxidant defenses, including GST, SOD, and CAT activity, together with Nrf2 activation in healthy flies (Fernandes et al., 2023). Similar biological effects were observed for free CUR only at concentrations above 300 μM (approximately 110.5 ng/mL). Therefore, the doses of 37 and 110 ng/mL were selected to ensure pharmacological relevance while maintaining safety during chronic exposure.

It is well established that the absorption, distribution, and elimination of xenobiotics in *D. melanogaster* share important similarities with humans (Mishra et al., 2022). Recently, *D. melanogaster* has been proposed as a novel model for pharmacokinetic investigations in healthy flies (Sadova et al., 2024). Sex-related differences were observed, with higher exposure in females, which was associated with differences in efflux transporter expression: the *Mdr50* gene in flies, orthologous to human *ABCB1* (P-glycoprotein), was more highly expressed in males, potentially leading to increased efflux of substrates. Age did not influence total isoflavone concentrations.

**FIGURE 5:**
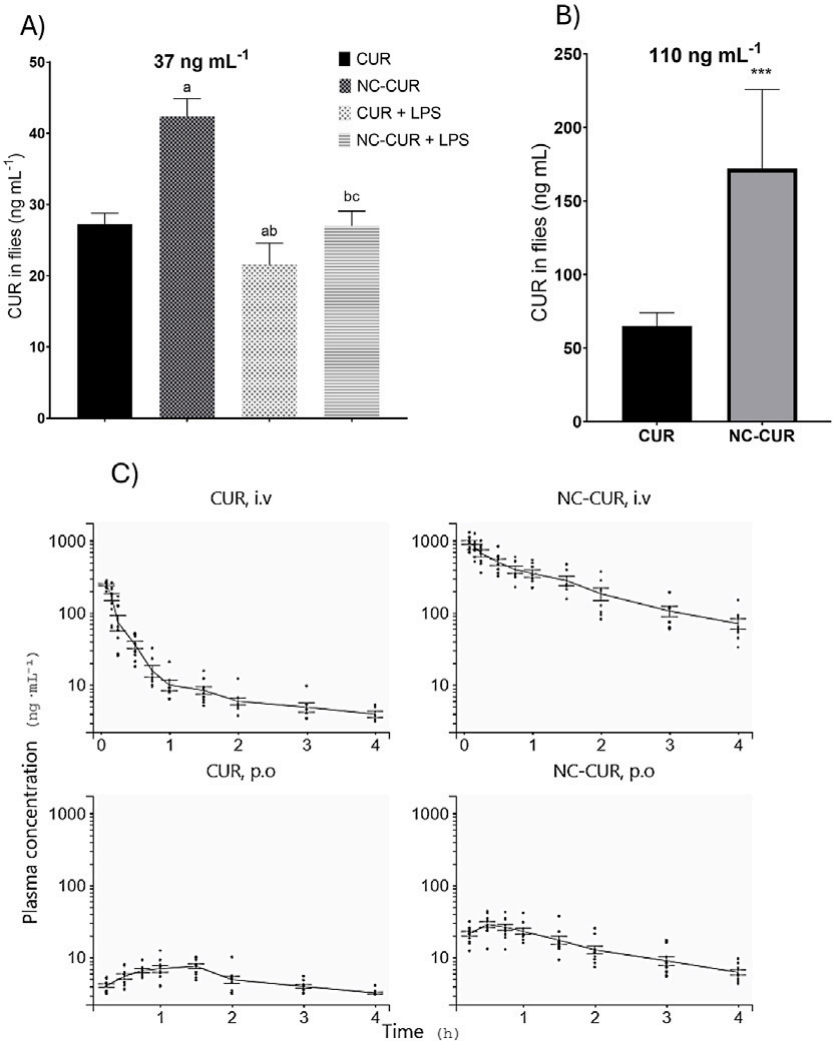
Data are presented as mean ± standard deviation (SD). **(A)** Quantifying CUR in whole-body homogenates of *D. melanogaster* exposed to CUR or NC-CUR at concentrations of 37 ng/mL or **(B)** 110 ng/mL for 10 days. **(C)** Pharmacokinetic profiles of male Wistar rats treated with CUR or NC-CUR, administered intravenously (2 mg/kg) or orally (6 mg/kg) (n = 9–12 animals per group). Significant differences are indicated as follows: ^*a*^ significant difference compared to the CUR group; ^*b*^ significant difference compared to the NC-CUR group; ^*c*^ significant difference compared to the CUR + LPS group (one-way ANOVA, followed by Tukey’s test. ^***^ *p* < 0.001 compared to the CUR group (unpaired Student’s t-test, n = 3 replicates).

Figure 5A displays the results of CUR concentration in *D. melanogaster* exposed to 37 ng/mL of either free CUR or NC-CUR under various conditions, including the presence of LPS as an inflammation inducer. In order to confirm that LPS exposure resulted in a systemic inflammatory state, a complementary set of analyses was performed, including behavioral assessment and quantification of oxidative stress biomarkers (reactive species levels, TBARS) and antioxidant enzyme activities (GST, SOD, and catalase). All parameters demonstrated significant alterations consistent with an inflammatory response, confirming the effectiveness of LPS-induced inflammation in this model (data not shown).

Our results indicate that flies treated with NC-CUR displayed the highest CUR concentrations, significantly surpassing those treated with free CUR (*p* < 0.0001), demonstrating that nanoencapsulation improves bioavailability and whole-body retention. LPS exposure markedly reduced CUR levels in flies treated with free CUR, whereas this effect was less pronounced for NC-CUR (*p* < 0.0001), indicating a protective influence of nanoencapsulation against inflammation-driven reductions in drug exposure. This co-exposure protocol reflects a preventive approach in which CUR and NC-CUR are administered from the onset of the inflammatory challenge, thereby modulating the early establishment and progression of inflammation.

Preliminary data also showed that NC-CUR improved survival in LPS-challenged flies compared with both LPS alone and free CUR + LPS groups (Supplementary Figure S2). The reduction in survival observed after day 7 is likely related to the progression of the inflammatory condition rather than aging, since age does not appear to significantly affect total exposure to xenobiotic biomolecules in *D. melanogaster* (John et al., 2022; Sadova et al., 2024).

These findings align with literature reports describing curcumin-mediated attenuation of LPS-induced inflammatory and oxidative damage in rodents, accompanied by improved antioxidant defenses (WeiGang et al., 2023; Seady et al., 2025). These findings corroborate the improved antioxidant activity previously described by our research group in healthy *D. melanogaster*, particularly through the increased expression of glutathione S-transferase, catalase, and superoxide dismutase in NC-CUR-treated flies, which achieved more significant responses at lower concentrations (Fernandes et al., 2023). Importantly, no differences in dietary intake were observed between treatment groups, supporting that increased CUR concentrations in NC-CUR groups were not related to food consumption (Fernandes et al., 2023).

The NC-CUR + LPS group exhibited a significantly lower drug concentration than the NC-CUR treated healthy flies (*p* < 0.0001) but maintained higher levels than the free CUR-treated group. This suggests that despite the presence of inflammation, nanoencapsulation offers partial mitigation against LPS negative impact on CUR concentration, affording relative drug protection. Due to CUR’s inherently low water solubility and substantial first-pass metabolism, oral absorption is naturally low. The presence of LPS and resultant intestinal inflammation could potentially exacerbate these limitations, further diminishing CUR’s bioavailability (Ghosh et al., 2020).

Strategies to enhance hydrophilicity, inhibit transporter-mediated efflux, and improve membrane fluidity have involved coating NC with polysorbate 80. The polyethylene oxide chains of polysorbate 80 form a hydrophilic layer on the NC surface, thus stabilizing them in aqueous environments and reducing aggregation (Nakama et al., 2020; Santos et al., 2021; de Oliveira Pacheco et al., 2022). This stabilization facilitates improved dispersion, permeation, and passive transport absorption, thereby enhancing *in vivo* performance (Dimer et al., 2014; Bu et al., 2017).

Figure 5B depicts CUR concentrations in *D. melanogaster* exposed to 110 ng/mL of free CUR and NC-CUR. The findings confirm significant differences between the two formulations, with the NC-CUR group exhibiting significantly higher CUR concentrations than the free CUR group (*p* < 0.001). While the CUR group exhibited concentrations around 75 ng/mL, the NC-CUR group reached approximately 200 ng/mL, highlighting that CUR nanoencapsulation resulted in significantly higher drug retention in the flies.

Additionally, our previous study indicated that flies chronically treated with NC-CUR showed an increased survival rate compared to control groups treated with saline, likely due to the upregulated expression of redox system-related enzymes (e.g., glutathione S-transferase and catalase) (Fernandes et al., 2023). These results indicate that the NC-CUR formulation promotes longer retention of CUR in *D. melanogaster*, even in the presence of LPS-induced inflammation. Although LPS reduces CUR concentrations in both free and encapsulated forms, the effect is less pronounced when CUR is nanoencapsulated, reinforcing the potential of nanocapsules to protect the drug and promote sustained release. Nanoencapsulation of bioactive compounds may also enhance absorption by limiting efflux via P-gp-type transporters, when such efflux is relevant. Although evidence suggests that CUR modulates P-gp activity and may be subject to efflux, the data remain inconclusive (Flory et al., 2021). This mechanism could partly explain why higher CUR concentrations were observed in the NC-CUR treated animals. Another plausible mechanism to explain the increased exposure observed with NC-CUR is the improved chemical stability and aqueous solubility afforded by nanoencapsulation (Michels et al., 2019; Liu et al., 2024).

#### 3.4.2 Pharmacokinetics

We conducted a pharmacokinetic study in healthy rats to elucidate the changes in exposure following chronic treatment with CUR and NC-CUR in flies. Pharmacokinetic analyses were performed after both intravenous and oral administration. Figure 5C illustrates the plasma pharmacokinetic profiles for each treatment, highlighting distinct differences in exposure, distribution, and absorption between CUR and NC-CUR across both administration routes.

Table 5 summarizes the pharmacokinetic parameters estimated through non-compartmental analysis, reinforcing the initial observations. For intravenous administration, the concentration-time curve from zero to infinity (AUC 0‐∞ ) for CUR was 100.41 ± 24.34 h ng/mL, while for NC-CUR, the AUC 0‐∞ was significantly higher at 1337.8 ± 385.18 h ng/mL (13.3-fold higher, *p* < 0.0001). Correspondingly, the clearance (CL) for CUR was 7.51 ± 1.81 L/h, whereas NC-CUR exhibited a markedly reduced CL of 0.57 ± 0.18 L/h (13-fold reduction, *p* < 0.0001), indicating a slower elimination rate. The elimination half-life (t_1/2_) also differed significantly, with CUR showing a t_1/2_ of 1.77 ± 0.45 h compared to 2.11 ± 1.02 h for NC-CUR (1.2-fold increase, *p* < 0.01).

**TABLE 5:** Pharmacokinetic parameters estimated by non-compartmental analysis.

Intravenous (n = 9)	CUR			NC-CUR		
	Mean	SD	CV (%)	Mean	SD	CV (%)
AUC0-∞ (h.ng/mL)	100.41	24.44	24.34	1337.8****	385.18	28.79
Cmax (ng/mL)	231.42	40.56	17.52	931.77****	187.8	20.16
kel (h−¹)	0.75	0.55	72.74	0.39*	0.15	39.04
t_1/2_ (h)	1.17	0.45	38.79	2.11**	1.02	48.2
MRT (h)	0.79	0.23	28.79	2.25***	1.22	54.28
CL (L/h)	7.71	1.81	23.49	0.57****	0.18	31.29
Vd (L)	12.81	6.17	48.18	1.66****	0.81	48.88
Oral (n = 12)						
AUC0-∞ (h.ng/mL)	25.55	7.17	28.07	82.23****	31.68	38.53
Cmax (ng/mL)	8.04	2.17	27.01	28.5****	8.65	30.36
Tmax (h)	1.31	0.28	21.68	0.58****	0.12	21.1
kel (h−¹)	0.54	0.38	69.69	0.32	0.11	33.91
t_1/2_ (h)	1.7	0.73	43.02	2.47**	0.91	36.85
MRT (h)	3	0.76	25.46	3.29	0.98	29.92
F_abs_	8.48			2.05		
F_rel_				321.84		

The results were expressed as means ± standard deviations (SD), followed by coefficients of variation (CV%) (n = 9–12 animals per group). An unpaired t-test was used for data with normal distribution, and the Mann-Whitney test was applied to non-normally distributed data. The pharmacokinetic parameters analyzed included the area under the plasma concentration-time curve from zero to infinity (AUC 0‐∞ ), clearance (CL), elimination half-life (t_1/2_), elimination rate constant (kel), mean residence time (MRT), volume of distribution (Vd), maximum plasma concentration (C_max_), time to reach maximum plasma concentration (T_max_), absolute bioavailability (Fabs), and relative bioavailability (F_rel_). Significance was denoted as follows: ^*^ *p*-value <0.05, ^**^ *p*-value <0.01, ^***^ *p*-value <0.001, ^****^ *p*-value <0.0001 when compared to the CUR group of each administration type.

The reduced elimination of CUR from nanoparticulate systems is associated with decreased opsonization, which involves interactions between circulating proteins and the particle surface. This allows NC to evade uptake by specialized reticuloendothelial system cells, leading to higher plasma exposure, prolonged circulation time, and slower elimination (Chen et al., 2017). A similar system for carrying highly metabolizable antipsychotic drugs has demonstrated an increased half-life in rats (Carreño et al., 2016b; 2016a; Vieira et al., 2016), attributed to reduced hepatic distribution, thereby reflecting a decrease in total clearance (Dimer et al., 2014; Carreño et al., 2016b; Carreño et al., 2016a). Given its physicochemical properties, the presence of polysorbate 80 as a coating in nanosystems exerts a steric effect that prevents macrophage uptake. Consequently, nanoencapsulated systems may also support more flexible and personalized dosing regimens, reducing the need for multiple administrations.

It has been noted that particles with diameters between 100 and 200 nm have longer circulation times compared to those larger than 250 nm (Liu et al., 1992). Additionally, our *in vitro* release data demonstrated that the sustained release profile of NC-CUR contributed to a longer residence time *in vivo* and likely a prolonged duration of action compared with free CUR. Similar observations have been reported in studies using poorly soluble and poorly bioavailable drugs, where nanoencapsulation resulted in sustained plasma exposure and controlled release behavior (Vieira et al., 2016; Funguetto-Ribeiro et al., 2023a).

The MRT was significantly longer for NC-CUR, with a mean value of 2.25 ± 1.22 h following intravenous administration, compared to 0.79 ± 0.23 h for CUR (*p* < 0.05). A notable reduction in the Vd was also observed in animals treated with NC-CUR, with a volume of distribution of 1.66 ± 0.81 L compared to 12.81 ± 6.17 L for CUR (7.7-fold reduction, *p* < 0.001). This reduction suggests a higher drug concentration in highly perfused organs when delivered via NC-CUR. Similar findings were observed in our previous study, where animals treated with nanoencapsulated clozapine showed substantial higher plasma exposure, decreased central compartment distribution, and improved apomorphine-induced stereotypy (Funguetto-Ribeiro et al., 2023a).

For oral administration, CUR demonstrated an AUC 0‐∞ of 25.55 ± 7.17 h ng/mL, while NC-CUR exhibited a much higher AUC 0‐∞ of 82.23 ± 31.68 h ng/mL (3.22-fold higher, *p* < 0.01). The maximum plasma concentration for CUR was 8.04 ± 2.17 ng/mL, whereas NC-CUR reached a maximum plasma concentration of 28.5 ± 8.65 ng/mL (3.54-fold higher, *p* < 0.01). Compared to CUR via the oral route, the relative bioavailability of NC-CUR was calculated to be 321.84%, indicating substantially better systemic exposure than the CUR solution. This increased bioavailability can be attributed to changes in disposition and improved absorption (Dimer et al., 2014; Carreño et al., 2016a; Han et al., 2024). The primary oral absorption mechanism of NC involves active transport mediated by microfold cells to the lymphatic system. Nevertheless, both paracellular and transcellular passive transport mechanisms also play a significant role and should not be overlooked (Han et al., 2024).

The faster absorption observed in animals treated orally with NC-CUR is reflected in the time to reach maximum plasma concentration, which was 0.58 ± 0.12 h for NC-CUR compared to 1.31 ± 0.28 h for CUR (0.44-fold; *p* < 0.05). Together, these pharmacokinetic changes induced by CUR nanoencapsulation help explain the increased exposure also observed in the flies. This finding is supported by evidence demonstrating relevant physiological similarities between mammalian and fly intestinal structures, particularly in cellular composition (Apidianakis and Rahme, 2011).

Most genes and metabolic pathways involved in hepatic detoxification in humans are conserved in *D. melanogaster*, enabling translational studies aimed at understanding pharmacokinetic behavior and drug metabolism in comparison with mammalian models, particularly considering that curcumin metabolism occurs predominantly in the liver. Overall, fundamental biological processes are highly conserved between insects and humans, with approximately 75% of human disease-related genes having homologs in *Drosophila*, while Wistar rats share about 80% genetic similarity with humans (Moraes and Montagne, 2021).

Similar to mammals, *D. melanogaster* exhibits analogous mechanisms of absorption, distribution, metabolism, and excretion (Mishra et al., 2022). More specifically, this species expresses Phase I and Phase II biotransformation systems responsible for xenobiotic metabolism, including reduction, glucuronidation, and sulfation, which are relevant pathways for the metabolism of polyphenols such as curcumin.

The pharmacokinetic profile obtained in rats provides important context to interpret the exposure patterns observed in both healthy and LPS-challenged flies, since rodents remain the gold standard preclinical model for systemic pharmacokinetic evaluation. The findings in *D. melanogaster* reflect exposure after chronic administration and should not be directly inferred to behave identically to rat pharmacokinitc outcomes, even though strong genetic homology and similar physiological processes between flies and mammals are well documented (Moraes and Montagne, 2021; Mishra et al., 2022; Sadova et al., 2024). By evaluating the same nanocarrier system in both species within a single study, we confirmed that the exposure trends observed in flies are consistent with what is expected based on mammalian literature.

Further work is needed to strengthen the translational relevance of *D. melanogaster* for pharmacokinetic applications. These include quantification of the free (unbound) fraction of CUR in rats, organ-specific exposure in flies, and measurements at multiple time points to characterize the full time course of CUR disposition. Such investigations will deepen our mechanistic understanding and help expand the use of *D. melanogaster* as an early screening model for nanoencapsulation strategies, including under pathological conditions.

## 4 Conclusion

This study developed and validated the first bioanalytical method capable of quantifying CUR in biological matrices from both flies and rats exposed to NC-CUR. The *in vitro* release profile confirmed the nanosystem’s capability for sustained drug release. *In vivo* studies showed that healthy and LPS-induced inflamed flies chronically treated with NC-CUR displayed higher overall exposure. Additionally, pharmacokinetic analysis in healthy rats demonstrated enhanced absorption and reduced elimination compared to free CUR, resulting in increased systemic exposure. Together, these results highlight the therapeutic potential of improving CUR retention while mitigating the detrimental impact of inflammation. Moreover, this work explicitly introduces a novel cross-species pharmacokinetic assessment framework, supporting the use of *D. melanogaster* as a complementary quantitative model before rodent studies. The strategy established here may enable more rational dose translation, streamline early screening of nanoformulations, and ultimately contribute to the progression of NC-CUR toward advanced preclinical and future clinical development.

## Data Availability

The raw data supporting the conclusions of this article will be made available by the authors, without undue reservation.
