# Supplementary material for: Improved exposure of curcumin-loaded nanocapsules: drug quantification in LPS-induced Drosophila melanogaster and pharmacokinetics in Wistar rats
Source: Front Pharmacol. 2025 Dec 1;16:1688992. doi: 10.3389/fphar.2025.1688992 (PMC12702928; doi:10.3389/fphar.2025.1688992)
Supplement: Supplementary file 1 [file Supplementaryfile1.docx]

Supplementary Material


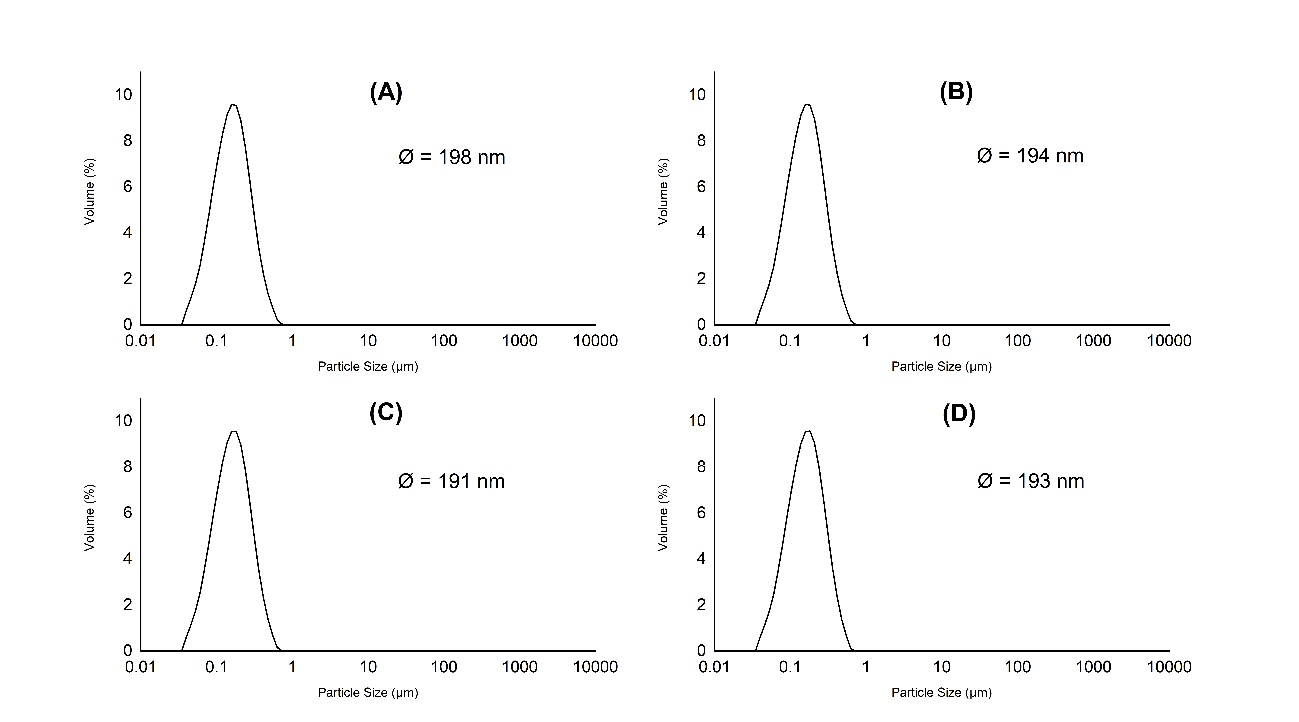


**Figure S1.** Volume-based size distribution of NC-CUR determined by DLS at different storage times: A) Day 1; B) Day 14; C) Day 21; and D) Day 30, after storage at ambient temperature (25 ± 5 °C).

**Figure S2.** Kaplan–Meier survival curves of Drosophila melanogaster treated for 10 days with saline (control), LPS, LPS + free CUR (37 ng/mL), or LPS + NC-CUR (37 ng/mL).
